# Transition in inherited metabolic diseases: the dietitians, pediatricians and adult physicians’ point of view: the results of an Italian survey

**DOI:** 10.1186/s13023-025-03755-8

**Published:** 2025-05-22

**Authors:** Alice Rossi, Chiara Pancaldi, Maria Giulia Regazzi, Giulio Agnelli, Valentina Assirelli, Antonio Barbato, Federico Baronio, Andrea Benso, Silvia Maria Bernabei, Giacomo Biasucci, Mara Botti, Cristina Bonfanti, Andrea Bordugo, Giulia Bruni, Alberto Burlina, Egidio Candela, Maria Teresa Carbone, Rosa Carella, Francesca Carubbi, Annalia Cianflone, Alessandra Cipriani, Silvia Coacci, Giuliana Da Prato, Sabrina De Leo, Valeria Di Natale, Alice Dianin, Carlo Dionisi Vici, Ilaria Fasan, Stefania Ferraro, Massimiliano Filosto, Serena Gasperini, Sara Giorda, Giorgia Gugelmo, Chiara Guzzetti, Concetta Latina, Christian Loro, Evelina Maines, Giacomo Marchi, Elena Massimino, Dorina Mita, Francesca Nardecchia, Davide Noto, Rita Ortolano, Sabrina Paci, Rossella Parini, Sara Parolisi, Giulia Paterno, Lidia Pontillo, Angela Pozzoli, Roberta Pretese, Sara Quattrini, Alice Re Dionigi, Valentina Rovelli, Simona Salera, Ferruccio Santini, Iris Scala, Annalisa Sechi, Claudia Sgattoni, Michele Stecchi, Alessandra Tavian, Antonio Toscano, Albina Tummolo, Maria Letizia Urban, Elena Verrecchia, Nicola Vitturi, Claudia Zuppaldi, Juri Zuvadelli, Lucia Brodosi

**Affiliations:** 1 Clinical Nutrition and Metabolism Unit, IRCCS AOUBO, Via Albertoni 15, 40138 Bologna, Italy; 2 Department of Medical and Surgical Sciences, University of Bologna, Via Zamboni 33, 40126 Bologna, Italy; 3 Pediatric Unit, IRCCS Azienda Ospedaliero-Universitaria di Bologna, Via Albertoni 15, 40138 Bologna, Italy; 4 Department of Clinical Medicine and Surgery, University of Naples “Federico II”, Via Pansini 5, 80131 Naples, Italy; 5 Division of Endocrinology, Diabetology and Metabolism, Department of Medical Sciences, University of Turin, Corso Dogliotti 14, 10126 Turin, Italy; 6 Department SITRA Nutritional Rehabilitation Unit, Bambino Gesù Children’s Hospital IRCCS, Piazza Sant’Onofrio 4, 00165 Rome, Italy; 7 Department of Medicine and Surgery, University of Parma, Via Gramsci 14, 43125 Parma, Italy; 8 Pediatrics and Neonatology Unit, Regional Referral Clinical Center for Inborn Errors of Metabolism, Regional Referral Clinical Center for Pediatric Eating Disorders, Lipigen National Pediatric Center, Guglielmo da Saliceto Hospital, Via Taverna 49, 29121 Piacenza, Italy; 9 Metabolic Rare Disease Unit, Pediatric Department, IRCCS San Gerardo Hospital, Via Pergolesi 33, 20900 Monza, Italy; 10 Regional Coordinating Centre for Rare Diseases, University Hospital of Udine, Piazzale Santa Maria Della Misericordia 15, 33100 Udine, Italy; 11 Dietetic Unit, Meyer Children University Hospital IRCCS, Viale Pieraccini 24, 50139 Florence, Italy; 12 Division of Inherited Metabolic Diseases, Reference Centre Expanded Newborn Screening, University Hospital Padova, Via Giustiniani 2, 35128 Padua, Italy; 13 UOSD Metabolic Diseases, AORN Santobono-Pausilipon, Via Fiore 6, 80129 Naples, Italy; 14 Department of Metabolic Diseases, Clinical Genetics and Diabetology, Giovanni XXIII Children Hospital, Azienda Ospedaliero-Universitaria Consorziale, Piazza Giulio Cesare 11, 70124 Bari, Italy; 15 Metabolic Internal Medicine Unit, University Hospital of Modena at Baggiovara, University of Modena and Reggio Emilia, Via Giardini 1355, 41126 Modena, Italy; 16 UOSD Diabetology, AST Fermo, Via Gigliucci 1, 63900 Fermo, Italy; 17 Division of Endocrinology, Diabetes and Metabolism, Department of Medicine, University Hospital of Verona, Piazzale Aristide Stefani 1, 37126 Verona, Italy; 18 Unit of Child Neurology and Psychiatry, Department of Human Neruroscience, University Hospital Policlinico Umberto I of Rome, Sapienza University of Rome, Via Dei Sabelli 108, 00185 Rome, Italy; 19 Pediatric Unit Regional Centre for Newborn Screening, Diagnosis and Treatment of Inherited Metabolic Diseases and Congenital Endocrine Diseases, Azienda Ospedaliera Universitaria Integrata di Verona, Piazzale Aristide Stefani 1, 37126 Verona, Italy; 20 Division of Metabolic Diseases and Hepatology, Bambino Gesù Children’s Hospital IRCCS, Piazza Sant’Onofrio 4, 00165 Rome, Italy; 21 Division of Clinical Nutrition, Department of Medicine, University Hospital, Via Giustiniani 2, 35128 Padua, Italy; 22 Pediatric Unit, Department of Health Sciences, Magna Graecia University of Catanzaro, Viale Europa, 88100 Catanzaro, Italy; 23 Department of Clinical and Experimental Sciences, NeMO-Brescia Clinical Center for Neuromuscular Diseases, University of Brescia, Via Nava 4, 25064 Brescia, Italy; 24 Department of Pediatrics, Metabolic Diseases, University of Torino, Via Verdi 8, 10124 Turin, Italy; 25 Division of Metabolic Diseases, Department of Medicine, University Hospital of Padova, Via Giustiniani 2, 35128 Padua, Italy; 26 SSD Endocrinologia Pediatrica e Centro Screening Neonatale, Ospedale Pediatrico Microcitemico “A. Cao”, Via Jenner 18, 09121 Cagliari, Italy; 27 Clinical Pediatrics and Pediatric Endocrinology and Diabetology, Azienda Ospedaliera Universitaria Policlinico “G. Rodolico-San Marco”, Via Sofia 78, 95123 Catania, Italy; 28 Division of Pediatrics, S. Chiara General Hospital, APSS Trento, Largo Medaglie d’oro 9, 38122 Trento, Italy; 29 Internal Medicine Unit and MetabERN Health Care Provider, University Hospital of Verona, Piazzale Aristide Stefani 1, 37126 Verona, Italy; 30 Department of Human Neuroscience, Sapienza University, Viale Dell’Università 30, 00185 Rome, Italy; 31 Department of Health Promotion, Maternal and Child Health, Internal and Specialized Medicine of Excellence “G. D. Alessandro” (PROMISE), University of Palermo, Piazza Marina 61, 90133 Palermo, Italy; 32 Department of the Women and the Child, Inborn Errors of Metabolism, San Paolo Hospital - ASST Santi Paolo e Carlo University of Milan, Via Antonio Di Rudinì 8, 20142 Milan, Italy; 33 Nutritional Center, Giannina Gaslini Pediatric Institute IRCCS, Via Gaslini 5, 16147 Genoa, Italy; 34 Department of Pediatrics, Polytechnic University of Marche, Piazza Roma 22, 60121 Ancona, Italy; 35 Clinical Department of Pediatrics, San Paolo Hospital, ASST Santi Paolo e Carlo, Via Antonio di Rudinì 8, 20142 Milan, Italy; 36 Regional Clinical Center for Expanded Newborn Screening, Fondazione IRCCS Ca’ Granda Ospedale Maggiore Policlinico, Via Sforza 35, 20122 Milan, Italy; 37 Endocrinology Unit, Obesity and Lipodystrophy Center, University Hospital of Pisa, Via Paradisa 8, 56124 Pisa, Italy; 38 Department of Maternal and Child Health, Federico II University Hospital, Via Pansini 5, 80131 Naples, Italy; 39 Medical Genetics and Rare Disease Coordination, Institute of Maternal-Infantile Sciences, Ospedali Riuniti, Presidio Torrette,Via Conca 61, 60126 Ancona, Italy; 40 SOSD Technical Health Care Professions - Dietitian and Regional Coordinating Centre for Rare Diseases, Azienda Sanitaria Universitaria Friuli Centrale, Piazzale Santa Maria Della Misericordia 15, 33100 Udine, Italy; 41 Department of Clinical and Experimental Medicine, ERN-NMD Center of Messina for Rare Neuromuscular Disorders, University of Messina, Piazza Pugliatti 1, 98122 Messina, Italy; 42 Department of Experimental and Clinical Medicine, University of Florence, Largo Brambilla 3, 50134 Florence, Italy; 43 Department of Aging, Orthopaedic and Rheumatological Sciences, Centre for Continuity of Care and Frailty, Fondazione Policlinico Universitario A. Gemelli IRCCS, Largo A. Gemelli 8, 00168 Rome, Italy; 44 Department of Translational Medicine, University of Naples “Federico II”, Via Pansini 5, 80131 Naples, Italy

**Keywords:** Adolescent health, Transition, Inherited metabolic diseases, Italian survey

## Abstract

**Background:**

Patients affected by inherited metabolic diseases (IMDs), through effective newborn screening and better clinical management, are living longer and have a lower burden of disease; this rises the challenge of properly taking life-long care of them as they age. This study aims to assess the Italian experience with the transition of patients affected by IMDs from pediatrician to adult care, focusing on the dietetic approach as well. For this purpose, a survey was created on REDCap® and distributed via email to the members of the “Dietetics and Nutrition Working Group” and “Inherited Metabolic Diseases in Adults Working Group” of “Italian IMD and Newborn Screening Society” (SIMMESN); dissemination was possible with the collaboration of MetabERN.

**Results:**

A total of 49 complete responses were collected-28 from medical doctors (MDs) and 21 from dietitians-from 35 different centers. Considering the MDs, 13 take care of pediatric patients; the remaining 15, with heterogeneous specialization, of adults with IMDs. Considering the dietitians, only 6 deal with IMDs patients as their full-time activity. Out of the 35 centers, 19 do not have a transition program (while 10/19 are already trying to implement it); the main barrier identified to the implementation voted by 42% of participants is represented by the lack of identification of a suitable facility. Considering the 16 centers that already have a transition program, the 2 main difficulties reported by 43% of participants were the lack of a psychologist for adult centers and the lack of specific training in IMDs on the adult service team; this last option was also the most voted by the dietitians (44%).

**Conclusions:**

The administered survey allows us to capture the state of transition programs in Italy, the lack of homogeneity in those centers that already have one, and the obstacles to developing a new program. What unequivocally emerged is the need for standardization of the transition program and for delineating a path to train MDs specialized in treating adult patients with IMDs, as well as dedicated dietitians.

**Supplementary Information:**

The online version contains supplementary material available at 10.1186/s13023-025-03755-8.

## Background

Inherited metabolic diseases (IMDs) are disorders caused by an enzyme defect in biochemical pathways affecting protein, lipid, carbohydrate metabolism, or impaired organelle function. Examples of IMDs include phenylketonuria (PKU), maple syrup urine disease (MSUD), urea cycle disorders (UCDs), lysosomal storage diseases (LSDs) such as Gaucher disease and Fabry disease, and mitochondrial disorders [1]. IMDs are complicated medical conditions, that often involve several organ systems [1]. Individuals with IMDs are complex patients who imperatively require integrated management by an experienced team in IMDs, including metabolic doctors, dietitians, nurses, and psychologists [2]. The specific needs of each IMD will determine the appropriate tailored treatment for each disease. The pediatrician is essential in coordinating the multidisciplinary team during the pediatric age because many IMDs are diagnosed early at birth or during infancy [3]. Dietitians play a fundamental role in treating IMDs: dietary therapy is often one of the primary treatments, regardless of age. It would be advisable for the metabolic team of each pediatric and/or adult center to have at least one dietitian trained and specialized in IMDs who can manage and support patients undergoing dietary treatment. Early identification and treatment of these pathologies significantly affect patients prognosis, improving life expectancy and preventing adverse outcomes. In addition, the correct nutritional approach, promptly began, prevents metabolic decompensation in these patients and its negative consequences for neuropsychological development, improving quality of life [4]. This is particularly relevant for small molecule disorders, for which dietary therapy is often the main treatment strategy; timely this is only possible through newborn screening (NBS) for those diseases for which it is provided. Aging from childhood into adulthood is characterized by substantial biological, psychological, and behavioral changes [5]; while this is true for the general population, it is even more pronounced in individuals with IMDs. Adolescence, in particular, often coincides with reduced adherence to nutritional and/or pharmacological therapy [6].

Since 2016, by law, n. 167/2016, Italy has outlined itself as a leader in expanded NBS in Europe. With the extended application of newborn screening and the continuous development and improvement of new treatments, patients affected by IMDs have significantly increased their life expectancy and getting out of the area of competence of the pediatric units that have traditionally treated them [7]. Importantly, just a few decades ago, adult patients with IMDs were rare, and that is why, globally, health services have yet to find the best management for these patients properly. A good transition program, from pediatric units to centers specific for adults, has been the subject of attention to ensure optimal patient management and continuity of care. The aim is to attenuate the psychological (and therefore clinical) impact of changes [8]. Only through a successful and gradual transition program patients with IMDs can become fully independent and capable of taking life-long care of their health [9]. As young adults transition into independent individuals, they take on the responsibility of managing their own healthcare, engaging directly with medical professionals, and making informed decisions about their treatment. This process should be actively supported by both healthcare providers and parents, who play a crucial role in fostering a seamless connection between the adolescent and the adult metabolic care team [10]. Recent work by the International Working Group on Gaucher Disease (IWGGD) in 2024 highlighted key aspects of the transition process in Gaucher disease type 1, emphasizing the importance of structured transition pathways and the need for multidisciplinary involvement to ensure optimal patient outcomes [11]. Several other studies and guidelines highlight best practices in transition care, emphasizing the need for structured, multidisciplinary approaches [12–14]. Nevertheless, transition in IMDs is still a challenge because both adult metabolic physicians and adult metabolic centers are rare, and specific training in treating IMDs in adult patients is lacking [15].

To collect data on the transition program in Italy from personnel who care for patients with IMDs, a survey was created and disseminated to Italian centers that take care of IMDs. The first aim of this survey was to assess the Italian experience with the transition of patients affected by IMDs from pediatric care to adult care. The second purpose was to assess how the transition for patients with IMDs is managed in Italy regarding the dietetic approach, including the perspective of dietitians, as to our knowledge, this is the first study to consider this aspect.

## Methods

### Study design

This was a descriptive cross-sectional observational study. The project was proposed by the Italian IMD and NBS Society (SIMMESN) [16], particularly by its “Dietetics and Nutrition Working Group” in collaboration with “Inherited Metabolic Diseases in Adults Working Group; to augment dissemination MetabERN (European Reference Network for Hereditary Metabolic Disorders) [17] was also engaged. The project was coordinated by dietitians and MDs treating adult IMDs patients at the Clinical Nutrition and Metabolism Unit, IRCCS AOUBO, Bologna, Italy.

The survey primarily covers key aspects related to the transition of IMD patients, including clinical management, challenges, and dietary approaches. The specific aim was to assess the current state of transition practices in Italy, identify gaps, and explore areas for improvement. At this time, we do not plan to conduct further research based on the study's outcomes.

### Participants and selection criteria

The survey was sent to all the adherents to the “Dietetics and Nutrition Working Group” and “Inherited Metabolic Diseases in Adults Working Group” of SIMMESN. MetabERN Italia centers were also engaged. Every center in Italy that treats IMDs is included in the aforementioned groups. In particular, the survey was addressed to pediatricians, adult physicians, and dietitians. Participation in the survey was voluntary.

### Ethical considerations

Since no individual patient data are reported, but only aggregated data provided as responses by healthcare professionals, ethical committee approval was not needed.

### Survey development and administration via REDCap

The survey was created in the Italian language on REDCap® [18] and the link was sent via e-mail (Additional file 1). The survey was sent for the first time in June 2022 and a second time in January 2023 to expand the sample of participating centers. At the end of January 2023, the survey collection was closed, and participants could no longer access the REDCap link.

The survey evaluated different topics related to the transition process, as shown in the supplementary material. From a structural point of view, the survey was composed of two parts: the first part collected general data from the center; the second part was not fixed and provided a set of questions that were dependent on the subject completing it. This was done so that, whether the person answering was a dietitian or an MD, it was possible to delve more accurately into their specific point of view. In particular, the survey was addressed to all physicians involved in the management of these diseases, but not nurses, as the role of a dedicated nurse for these conditions has not yet been officially recognized in Italy. Moreover, physicians responded on the basis of their experience with all IMDs, whereas dietitians provided answers only regarding conditions that involve dietary management.

Most of the questions had closed-ended answers. The data (mean ± SD for continuous data) were reported using Microsoft Excel.

To better identify the subgroups of IMDs included, we referred to the categorization reported in Attachment 7 from the Decree of 12 January 2017 of the President of the Council of Ministers [7]. A CHERRIES checklist has been produced to improve the quality of survey reporting [19].

## Results

**Fig. 1:**
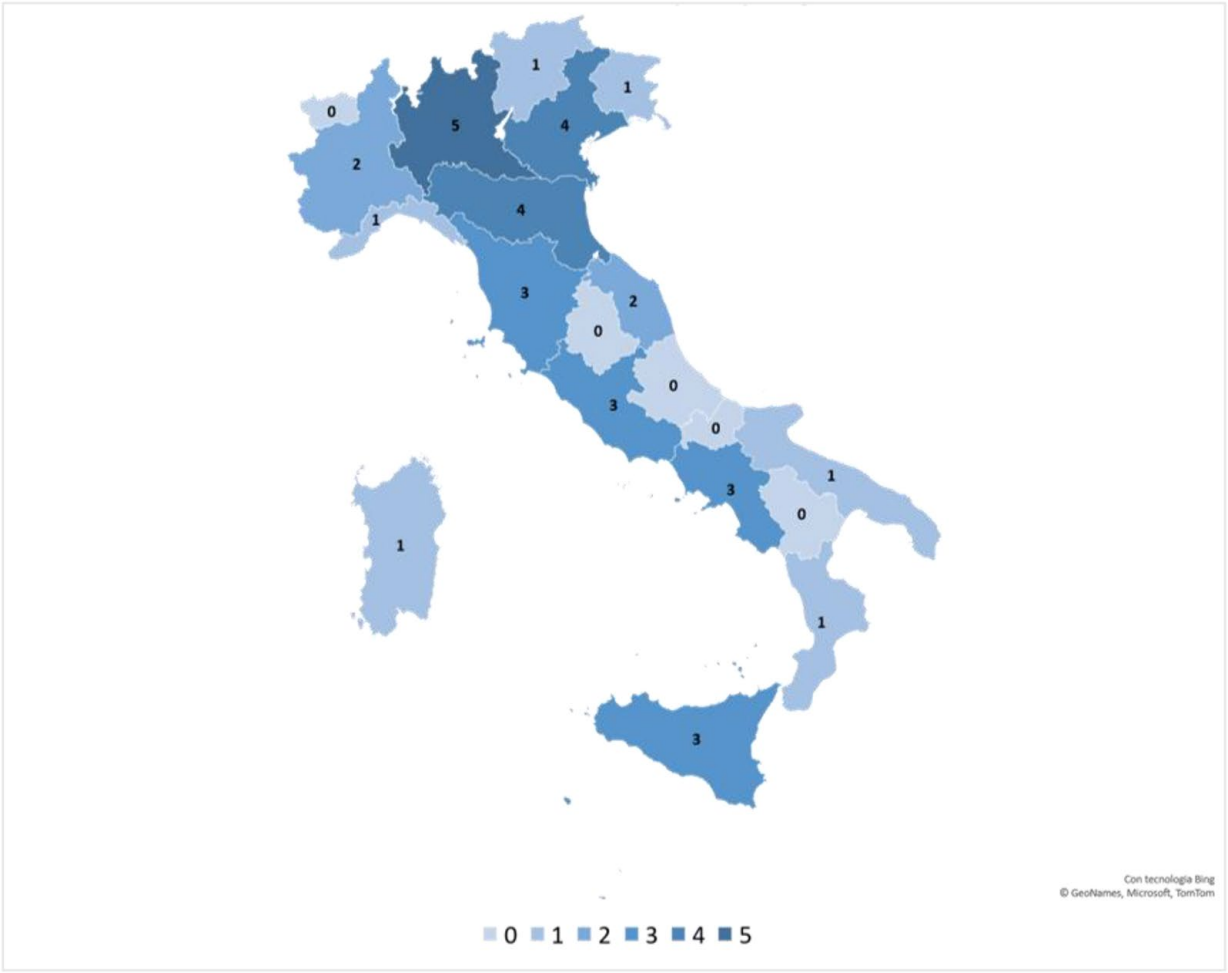
Number and distribution of the participating centers for each Italian region

A total of 49 complete responses were collected, 28 from MDs and 21 from dietitians, belonging to 35 different centers (Fig. 1).

**Fig. 2:**
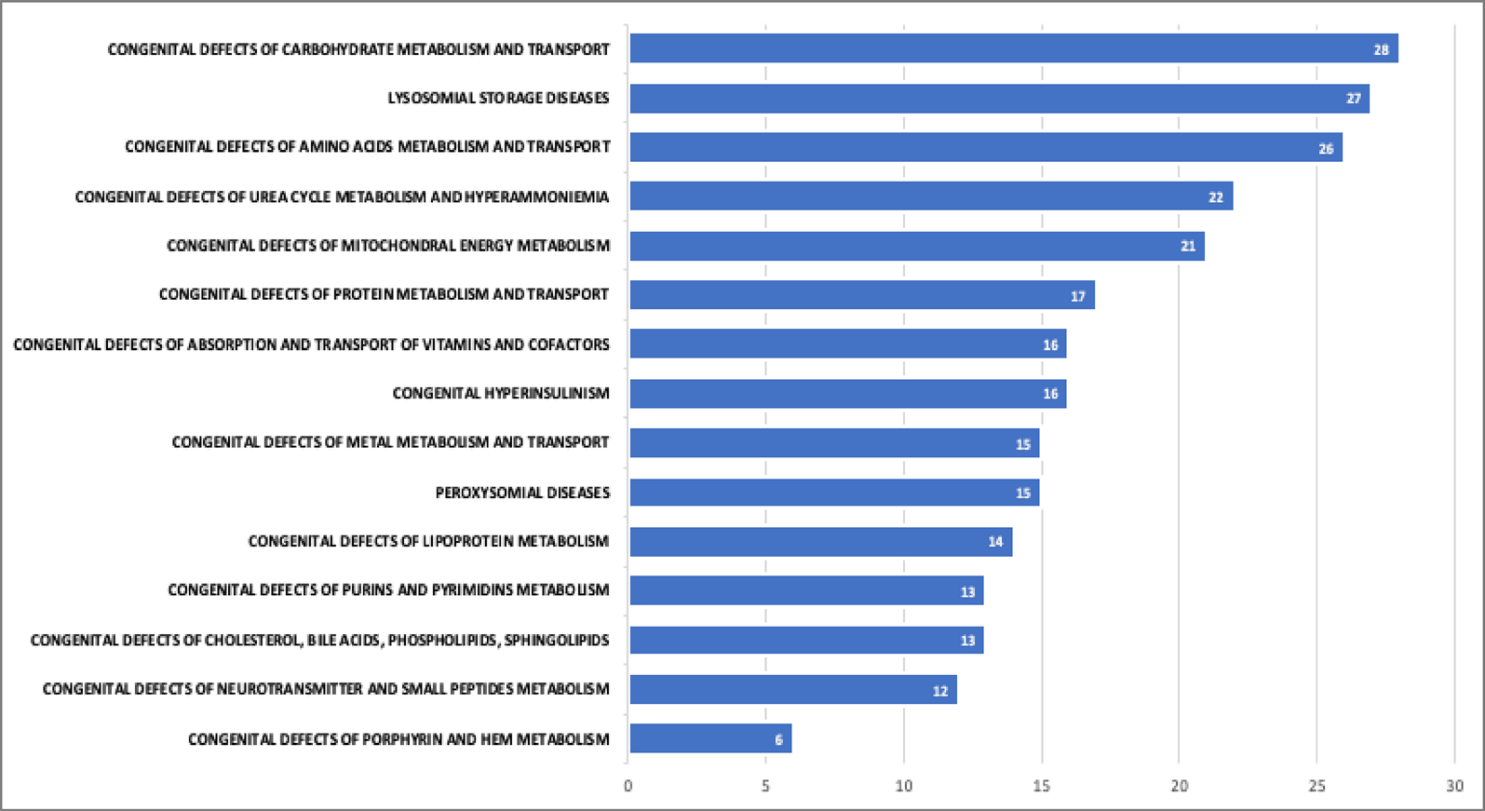
IMDs treated by the participating centers (the values represent the number of centers treating each IMD)

Of the 28 MDs who completed the survey, 12 were Pediatricians and 1 was specialized in Infantile Neuropsychiatry. The remaining 15 were physicians with various specializations: neurology (n = 2), endocrinology (n = 3), internal medicine (n = 7), genetics (n = 1), and nutrition (n = 2) (Fig. 2).

The professional area of expertise of dietitians was also investigated. Among the 21 dietitians, only 6 (29%) were dedicated exclusively to treating IMDs, while 15 dietitians (71%) also cared for patients with other conditions.

None of the centers involved treat all the listed diseases. Figure 2 shows the different IMDs treated by the centers.

The centers cared for patients aged between 0 and 87 years. The average age was not investigated.

Considering all the centers (n = 35), 16 reported having a transition program (institutional or not).

### Centers with an existing transition program

Among the 16 centers with a transition program, only 8 had a standardized transition protocol—a written document formally approved within the hospital explaining the methods and purposes of the transition process.

Regarding the transition process, among the 16 centers that have one, 7 are "sending centers,” and 9 are "receiving centers.”

On average, the centers have had the program active for six years (± 4.5 SD), with a median of 5 [2–15 years].

On average, 12.7 patients transit to adult care each year (range: 1–75). A maximum of 75 was reported by a center that had just started the transition. Excluding this answer, the adjusted average number of patients transferred/year was 6.2 (range: 1–10).

For 14 centers, the transition starts at a minimum age of 18, whereas in 1 center it starts at the age of 16; 1 center has yet to establish a designated age.

Excluding 1 center, all the centers carrying out a transition process held at least one joint visit in the presence of the pediatrician and the doctor treating adult patients. The average number of transition visits is 2 (1–10).

Of the 7 “sending centers” with a transition process, 5 centers refer their patients to the same hospital; in the same hospital, they might be referred either to a single department or to multiple departments on the basis of the patient’s disease. The other 2 centers transition their patients to a different hospital, and they might be referred either to a single department or to multiple departments on the basis of the disease.

Of the 5 centers that transition patients within the same hospital, 2 refer them to a single department (Clinical Nutrition, Rare Disease Coordination Center), and 3 refer them to more than one department each (Endocrinology, Internal Medicine, Neurology, Nephrology, Rare Disease Center). The remaining 2 centers refer their patients to other hospitals.

On the other hand, of the total number of "receiving centers” (n = 9), 7 received patients from a single or multiple pediatric facilities of the same hospital, whereas only 2 received them from a single pediatric facility of another hospital.

Centers with a transition program report that the team that treats adult patients after transition is composed of a doctor specialized in one of the following: Endocrinology (n = 4), Genetics (n = 2), Internal Medicine (n = 5), Nephrology (n = 1), Neurology (n = 2), Nutrition (n = 1), and missing data (n = 1). In addition, each team consists of a dietitian (14/16), a psychologist (8/16), a nurse (7/16), and a biologist (3/16).

**Fig. 3:**
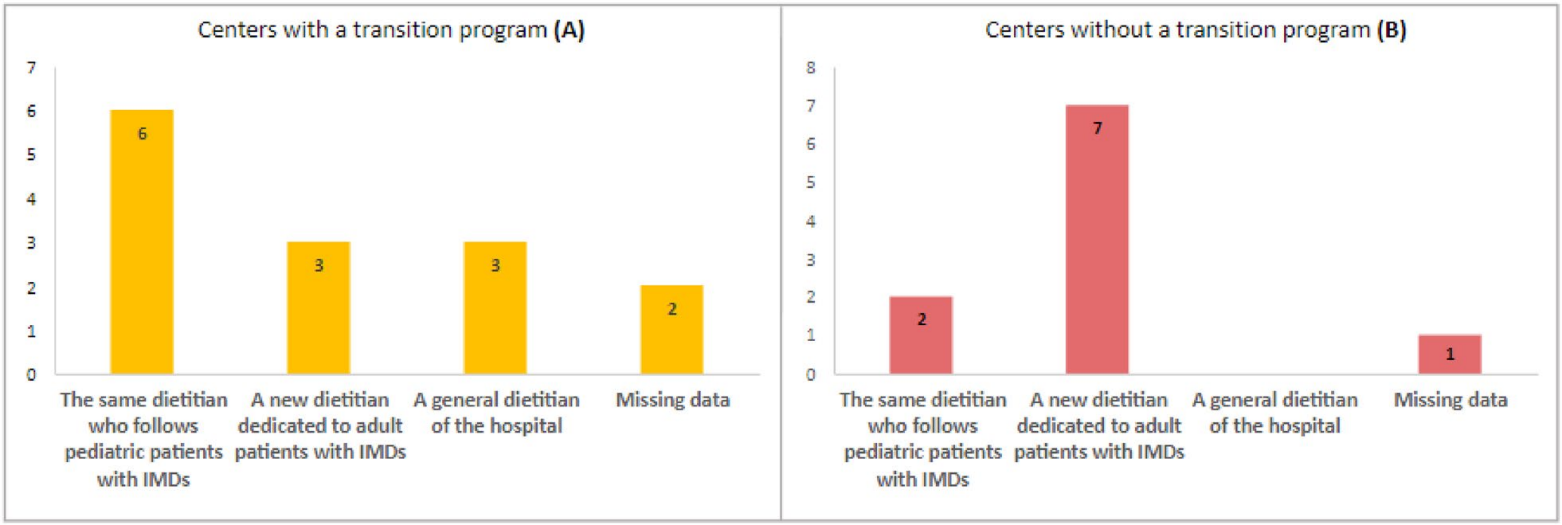
Nutritional management of adult patients with IMDs: comparison from centers with an active transition program (**a**) and centers currently without an existing transition program (**b**). Chart A shows which dietitian is entrusted with nutritional follow-up after transition. Chart B shows the dietitian who would be chosen if a transition program were to be started

Among the 14 centers with a dietitian in the team treating adult patients, in 6 centers the nutritional follow-up is carried out by the same dietitian who follows pediatric patients; in 3 centers a different dietitian dedicated to adult metabolic patients is present and in 3 centers by any dietitian in the clinical nutrition service. Data are missing for 2 centers. Figure 3a shows the comparison between the data described above and the responses of centers without a transition program.

A total of 5 out of 16 centers with a transition program, declared that a psychological and social assessment before the transition was performed, whereas 8 did not carry out it. Data are missing for the remaining 3 centers. Patient and family satisfaction was reported as a regular evaluation in 3 centers, whereas 10 did not evaluate the patients' opinions. Data are missing for 3 centers. Only 1 center evaluated the patient's quality of life a few months after the transition, whereas 12 reported not collecting those data when the survey was completed. Data are missing for 3 centers.

The survey also allows the collection of data on positive and negative logistical and organizational aspects. Tables 1 and 2 report the investigated items and answers from dietitians, adult physicians**,** and pediatricians.

**Table 1:** Positive aspects from a logistical and organizational point of view

Category	Dietitians N (%)	Adult physicians N (%)	Pediatricians N (%)	Total N (%)
Adequate information transfer between teams and/or facilities/OUs and access to patient data	6/9 (67)	7/9 (78)	5/5 (100)	18/23 (78)
Adequate management of the specialist skills of each professional (e.g. the pediatrician no longer deals with adult patients)	3/9 (33)	7/9 (78)	2/5 (40)	12/23 (52)
Treatment setting suitable for adult patients (e.g. presence of spaces/instruments suitable for adult patients)	4/9 (44)	5/9 (56)	2/5 (40)	11/23 (48)
Facilitated care path (booking of exams, consultations…)	3/9 (33)	4/9 (44)	2/5 (40)	9/23 (39)
Adequate laboratory resources/specific programs for patient follow-up	0	3/9 (33)	2/5 (40)	5/23 (22)
Others	0	0	1/5 (20)	1/23 (4)

Answers from 23 responders from 16 centers with a transition program: 9 dietitians, 9 adult physicians, and 5 pediatricians

**Table 2:** Logistical and organizational difficulties or obstacles

Category	Dietitians N (%)	Adult physicians N (%)	Pediatricians N (%)	Total N (%)
Sending patients to another facility (e.g. switch to a different hospital with a different location)	1/9 (11)	2/9 (22)	1/5 (20)	4/23 (17)
Difficulties in accessing patient data and medical history	0	0	0	0
Lack of a psychologist in the Adult Service	3/9 (33)	4/9 (44)	3/5 (60)	10/23 (43)
Lack of a dietitian in the Adult Service	3/9 (33)	2/9 (22)	4/5 (80)	9/23 (39)
Difficulties in transferring information between teams and/or facilities/departments	2/9 (22)	1/9 (11)	0	3/23 (13)
Pediatrician reluctant to refer the patient to another physician/facilities	1/9 (11)	2/9 (22)	0	3/23 (13)
Lack of specific training/experience on IMDs in the Adult Service team	4/9 (44)	3/9 (33)	3/5 (60)	10/23 (43)
Lack of appropriate care setting for adult patients (e.g. lack of space, instruments suitable for adult patients)	3/9 (33)	2/9 (22)	4/5 (80)	9/23 (39)
Lack of specific laboratory resources for patients follow-up (e.g. lack of a specialized testing laboratory, test booking programs..)	3/9 (33)	2/9 (22)	1/5 (20)	6/23 (26)
Others	1/9 (11)	3/9 (33)	1/5 (20)	5/23 (21)

Answers from 23 responders from 16 centers with a transition program: 9 dietitians, 9 adult physicians, and 5 pediatricians

Our survey did not collect detailed information on the transition models used in the two centers that reported having a transition protocol. Therefore, we are unable to provide specifics on how their transition clinics operate, the frequency of patient visits before the transfer of care, or the tools used to assess transition readiness. Similarly, we do not have data on how pediatric records are summarized before transfer to adult care. 

### Centers without an existing transition program

Among the 19 centers without an existing transition program, 13 treat both pediatric and adult patients; 2 pediatric centers declare extending the follow-up period for patients who are already being treated even beyond the age of 18; 4 centers reported caring for adult patients exclusively.

Among these 19 centers, 18 reported being favorable to the creation of a transition program. Of these, 10 declared that a transition program is already being planned to be activated in the future, whereas 8 reported that the activation of a transition program was not feasible; only 1 center reported that a transition program is not being considered at the time of the survey administration.

The survey collected data on the centers’ point of view on the potential need for a transition program: all but 1 center believed that it was necessary to establish a transition program for all the IMDs.

Of the 18 centers that reported being favorable for instituting a transition program, 6 centers reported that the transition should start at age 16, whereas 7 centers would begin at age 18; 1 center deemed it is necessary to evaluate the proper transition age on the basis of the IMD; 1 center defered to the general practitioner’s decision; 3 centers did not indicate a minimum age.

Of these 18 centers, 8 would transition patients to the same hospital; in the same hospital, they might be referred either to a single department or to multiple departments on the basis of the disease; 5 centers would transition patients to single or multiple departments in a different hospital. Data are missing for 5 centers.

The participating dietitians were asked who they would entrust with nutritional follow-up of adult patients when starting a transition program: 7 identified a new dietitian dedicated to IMDs, and 2 would entrust the same dietitian involved in pediatric patient management. None of them indicated a general dietitian of the hospital. Data are missing for 1 center. See Fig. 3b.

The survey collected data on the main difficulties in maintaining follow-up for both pediatric and adult patients from dietitians and pediatricians and collected data on the main barriers to the implementation of a transition program. Tables 3 and 4 summarize the investigated items and the answers from dietitians, adult physicians and pediatricians.

**Table 3:** Difficulties in maintaining follow-up for both pediatric and adult patients

Category	Dietitians N (%)	Pediatricians N (%)	Total N (%)
Growing number of patients and workload	7/12 (58)	6/8 (75)	13/20 (65)
Fewer experience on the nutritional aspects of the adult patient	2/12 (17)	1/8 (13)	3/20 (15)
Lack of an adult specialist physician	4/12 (33)	3/8 (38)	7/20 (35)
Organization of specific care pathways for the adult patient (e.g. enzyme replacement therapy, pregnancy..)	4/12 (33)	6/8 (78)	10/20 (50)
Others	1/12 (8)	0	1/20 (5)

Answers from 20 responders from 13 centers without a transition program, that followed both pediatric and adult patients: 12 dietitians and 8 pediatricians

**Table 4:** Barriers to the implementation of a transition program

Category	Dietitians N (%)	Adult physicians N (%)	Pediatricians N (%)	Total N (%)
Identification of a suitable facility/Operating Unit to which patients (from the same or a different hospital) can be transited	6/12 (50)	0	5/8 (63)	11/26 (42)
Lack of one or more dedicated professionals (e.g. physician, dietician, psychologist, nurse..)	7/12 (58)	1/6 (17)	0	8/26 (31)
Difficulty in identifying all the equipment, spaces, and resources needed for patient care	4/12 (33)	1/6 (17)	1/8 (13)	6/26 (23)
Others	1/12 (8)	3/6 (50)	1/8 (13)	5/26 (20)

Answers from 26 responders from 19 centers without a transition program: 12 dietitians, 6 adult physicians, and 8 pediatricians

## Discussion

The transition of patients affected by IMDs is a pressing topic, as patients with IMDs currently live longer and have better quality of life. This presents new challenges, as the management of adult patients affected by IMDs and possible complications is not always well defined by specific guidelines or evidence-based medicine but is often based on the experience of centers [20].

The European Reference Network for Hereditary Metabolic Disorders (MetabERN) disseminated a survey on transition programs through 63 centers of MetabERN from 20 countries in Europe [21]. The main challenges identified were the lack of time and the shortage of MD experts in treating adults with IMDs. Conversely, the keys identified for a successful transition program include the presence of a transition coordinator, medical staff entirely dedicated to transition, and targeted training for physicians [21]. An Italian group of clinicians treating PKU published an expert opinion on transition in patients with PKU, in 2022, hightlighting similar critical points [22].

Comparing our findings with those from other countries, the study by Stepien et al. [23] provides valuable insights into the challenges faced in the United States regarding the transition of IMD patients. The main barriers reported in the U.S include the lack of structured transition protocols, insufficient knowledge of IMDs among adult healthcare providers, and disparities in transition readiness due to social and legal differences. These challenges closely mirror those identified in Italy, particularly the limited number of specialized adult metabolic physicians and the absence of standardized transition programs. However, the U.S has seen some progress with the development of transition clinics, multidisciplinary transition teams, and readiness assessment tools such as the Ready Steady Go program. These initiatives aim to ensure a smoother transition process and might serve as a model for improving transition strategies in Italy. Future efforts in Italy should explore the feasibility of implementing similar structured programs to enhance the transition process and ensure better continuity of care.

Although there is a National Coordination Center that has the task of supervision, currently, in Italy, each center is organized individually, without clear guidelines, and without the allocation of specific funds from the government. A total of 16 centers have managed to set up a transition path; 8 have a transition program regulated by an officially approved document in the hospital, which has been ongoing for a range varying between a minimum of 5 and a maximum of 15 years. Among the centers that do not have a transition program, 18 out of 19 expressed interest in establishing one, despite facing individual difficulties and challenges due to the absence of a standardized system. This lack of standardization creates many difficulties, both in creating centers specialized in care for adult patients with IMDs and in creating a structured transition program. Undoubtedly, this can discourage centers treating adult patients from even trying to start a path. A panel of Italian experts produced 8 practical guidelines to obtain an adequate transition program for PKU patients, which could be especially beneficial to centers that are yet to develop one [22].

Even in the centers involved in the management of adult patients with IMDs, there is no homogeneity of care in terms of referred specialized MDs. This aspect underlines the importance of establishing a standardized and shared training path to offer patients the expected standardized quality of care. It is reasonable to assume that transitions may vary depending on the type of disease, given their differing care needs (e.g., risk of decompensation or not). However, addressing each disease group individually would have significantly complicated the study and made the survey more difficult to manage for each specific condition.

One of the major challenges in improving transition programs is the education and training of adult healthcare providers on rare metabolic diseases. Currently, structured educational programs specific to IMDs in adults are limited in Italy, and there is no standardized national curriculum. Training opportunities are often dependent on individual institutions or international collaborations. The development of a dedicated training pathway for adult physicians specializing in IMDs is crucial to ensure continuity of care and proper disease management.

The lack of homogeneity in MDs treating adult patients with IMDs, adversely affects the implementation of effective transition programs. In pediatric centers, this often determines referring patients to various specialists based on their predominant clinical manifestations, rather than to a physician who can serve as a central coordinator, engaging different specialists as needed based on the diverse clinical presentations of IMDs.

Another critical aspect is the funding of transition programs. At present, there are no designated governmental funds allocated specifically for transition services in IMDs in Italy [24]. Funding, when available, often comes from institutional hospital budgets, research grants, or patient advocacy groups [24]. Future efforts should aim to secure stable funding from national healthcare systems, European Union health programs, and philanthropic organizations to establish and sustain structured transition pathways. In Italy, Law 175/2021 assigns responsibility to reference centers for defining a personalized care pathway. This pathway includes the necessary treatments and monitoring for individuals affected by rare diseases, while also ensuring a structured transition from pediatric to adult care. However, practical implementation of this personalized care pathway is lacking.

In 14 out of the 16 centers with a transition program, patients transition at 18, which is the age of consent in Italy. Reviewing the literature, an agreement about the ideal age to start transitioning is yet to be found. Despite the need to agree on age, transition in patients with IMDs should be considered a process rather than a passage, which should occur over an adequate period to give the patient and the family the chance to accommodate the new care setting. To make this process effective, patients (and their respective families) must be informed and made aware early on by the pediatric unit that, as adults, they will be transitioned to a specific center that can better suit the particular needs of their disease as they grow older [25]. The number of joint visits combining both teams should be based on clinical needs and the organization of the centers (1–10).

The extreme variability of the transition paths in Italy is also reflected by the different compositions of the teams caring for adult patients. An MD, even with a different specialty, is always present; dietitians are not always present; dedicated nurses or psychologists are present in only half of the centers; and only 1 center reported having a dedicated biologist to organize and coordinate the transition process.

Considering sociopsychological aspects, only one third of the centers declared routine evaluations of their patients before starting the transition. Unfortunately, the survey did not delve further into the methods used, so we do not know how these centers evaluate these aspects. In 3 centers the degree of patient satisfaction with the transition process is routinely assessed; unfortunately, we do not have data related to the outcome of these evaluations, so at this time, we cannot comment on this matter. All the centers agreed that sharing clinical information for each transitioned patient between pediatric and adult centers efficiently and effectively was feasible, that the skills of the various MDs were recognized in this process, and that adult patients felt better in an environment tailored to their needs. Among the difficulties in maintaining follow-up for both pediatric and adult patients, the main concern that emerged was the lack of a doctor specialized in IMDs to treat adult patients. This again underlines the lack of standardization in the quality of care offered to patients. In addition, there is a lack of psychologists and a lack of space to create specialized clinics.

Among the 19 centers that do not have a transition program, all but one would replicate the pediatric management model, creating an adult center that cares for all IMDs regardless of specific clinical manifestations. These centers identify the desirable age to start the transition at 18, agreeing on what emerged from the centers with a program. Notably, 10 out of 19 centers are already working to start a transition program soon; with these centers, those with a transition program will potentially outnumber those that do not.

The high rate of adults lost to follow-up is another crucial element to be considered, among other reasons, to highlight the necessity of developing a transition program [21]. In fact, out of the 19 centers, only 6 reported losing less than 10% of adult patients. Having a specialized center for adult patients with IMDs, with standardized procedures, personnel, infrastructure, and a program to transition patients from pediatric units to adult units, would promote adequate standardized quality of care, consequently promoting adherence to treatment and medical follow-up of adult patients. In reference to Fig. 3a, it appears that in 6 out of 14 centers with a transition program, the same dietitian who follows pediatric patients with IMDs continues the follow-up for adult patients with IMDs as well. Considering 2 missing data points, 50% of these centers retain the same dietitian for both pediatric and adult patients with IMDs.

On the other hand, Fig. 3b shows that in centers without a transition program the majority of dietitians [7] would assign the nutritional follow-up of adult patients with IMDs to a new dedicated dietitian, whereas only 2 dietitians would maintain nutritional follow-up with the same pediatric dietitian.

As already highlighted, the presence of NBS and the increased life expectancy of patients with IMDs result in a larger number of adult patients in follow-up, leading to an increased workload and the need for specialized skills. This aspect is also relevant among the dietitians and physicians who participated in the survey. In fact, as shown in Tables 2 and 3, the lack of dedicated and trained dietitians for the treatment of adults with IMDs is identified as one of the main issues, both for those with an active transition program and for those without one.

Another relevant aspect is that MDs and dietitians working in centers where there is no transition are concerned with the growing workload and the difficulties in managing typical adult conditions, such as pregnancy. Once again, the lack of standardization in Italy is a critical point in establishing a transition program, as the main difficulties that hinder standartization of adequate structure and personnel trained in IMDs.

The strength of this study is the inclusion of all the main players in treating patients with IMDs, including dietitians and MDs for both pediatric and adult patients; the main aim is to provide, for the first time, an overview of the state of the transition programs in Italy, especially including centers that still do not have a program. Among the limitations of the study, the methodological one. Since this is a survey, the highest level of evidence is the participants' experience. Furthermore, not all doctors who deal with IMDs participated in this study.

## Conclusions

This survey captures the state of the transition programs from pediatric to adult centers for patients by IMDs in Italy, allowing a comparison of the existing programs and the reported difficulties in implementing them. Different centers share the same pivotal needs and problems, such as identifying an MD for adult patients and the lack of infrastructure, in the context of the various heterogeneous experiences specific to different centers. This particular phenomenon of similar needs, despite different center experiences, is secondary to the lack of standardization, which leaves each center to address joint problems differently. The need for standardization emerges most strongly in the absence of formal qualifications of the personnel who should treat adult patients with IMDs and in the lack of a formal and precise procedure on how a transition program itself should be structured. The poor availability of dietitians dedicated to adult metabolic services should be addressed in the future to improve both the transition processes and quality of care, considering the fundamental role of this figure in the management of various IMDs; once again, this poor availability can be traced back to the lack of standardization. Treating IMDs is a challenge, as they constitute a heterogeneous family of a large number of diseases with various clinical repercussions; in addition, the number of identified, diagnosed, treatable diseases is steadily growing. Consequently, considering both the ever-growing number of patients and how they are aging, with the needs specific to IMDs and the typical clinical and psychological problems of adulthood, it is necessary to act on the recognition of "metabolic medicine" as a subspecialty, with a defined training program and defined competencies; this is a necessity yet to be adequately met, both in Italy and all over the world.

Future efforts should focus on developing structured training programs for adult clinicians, fostering interest in IMDs, and securing funding for dedicated metabolic centers. Collaboration with national health policies, advocacy groups, and professional societies will be key in these efforts. Standardized guidelines should be developed by national and international metabolic disease organizations with input from multidisciplinary teams.

## Supplementary Information

Additional file 1.

## Declarations

### Availability of data and materials

The datasets used and/or analyzed during the current study are available from the corresponding author upon reasonable request.

## References

[1] Agana M, Frueh J, Kamboj M, Patel DR, Kanungo S. Common metabolic disorder (inborn errors of metabolism) concerns in primary care practice. Ann Transl Med. 2018;6(24):469. 30740400 10.21037/atm.2018.12.34 PMC6331353

[2] van Wegberg AMJ, MacDonald A, Ahring K, Belanger-Quintana A, Blau N, Bosch AM, et al. The complete European guidelines on phenylketonuria: diagnosis and treatment. Orphanet J Rare Dis. 2017;12(1):162. 29025426 10.1186/s13023-017-0685-2 PMC5639803

[3] Loeber JG, Platis D, Zetterstrom RH, Almashanu S, Boemer F, Bonham JR, et al. Neonatal screening in Europe revisited: an ISNS perspective on the current state and developments since 2010. Int J Neonatal Screen. 2021;7(1):15. 33808002 10.3390/ijns7010015 PMC8006225

[4] Grosse SD, Thompson JD, Ding Y, Glass M. The use of economic evaluation to inform newborn screening policy decisions: the Washington state experience. Milbank Q. 2016;94(2):366–91. 27265561 10.1111/1468-0009.12196 PMC4911729

[5] Chulani VL, Gordon LP. Adolescent growth and development. Prim Care. 2014;41(3):465–87. 25124201 10.1016/j.pop.2014.05.002

[6] White PH, Cooley WC, Transitions Clinical Report Authoring G, American Academy Of P, American Academy Of Family P, American College Of P. Supporting the health care transition from adolescence to adulthood in the medical home. Pediatrics. 2018;142(5). 10.1542/peds.2018-2587 30348754

[7] [ISS. Law 167/2016.]. https://www.iss.it/screening-neonatali/-/asset_publisher/BheO9tMYPdo8/content/10-screening-neonatale

[8] Sestini S, Paneghetti L, Lampe C, Betti G, Bond S, Bellettato CM, et al. Social and medical needs of rare metabolic patients: results from a MetabERN survey. Orphanet J Rare Dis. 2021;16(1):336. 34344397 10.1186/s13023-021-01948-5 PMC8329639

[9] Blum RW, Garell D, Hodgman CH, Jorissen TW, Okinow NA, Orr DP, et al. Transition from child-centered to adult health-care systems for adolescents with chronic conditions. A position paper of the Society for Adolescent Medicine. J Adolesc Health. 1993;14(7):570–6. 8312295 10.1016/1054-139x(93)90143-d

[10] Stepien KM, Hendriksz CJ. The principles of the transition process from paediatric to adult services in inborn errors of metabolism–own experience. Dev Period Med. 2015;19(4):523–7. 26982766

[11] Stepien KM, Znidar I, Kiec-Wilk B, Jones A, Castillo-Garcia D, Abdelwahab M, et al. Transition of patients with Gaucher disease type 1 from pediatric to adult care: results from two international surveys of patients and health care professionals. Front Pediatr. 2024;12:1439236. 39346636 10.3389/fped.2024.1439236 PMC11430091

[12] Fair CD, Sullivan K, Gatto A. Best practices in transitioning youth with HIV: perspectives of pediatric and adult infectious disease care providers. Psychol Health Med. 2010;15(5):515–27. 20835962 10.1080/13548506.2010.493944

[13] Cotts TB. Transition of care in congenital disease: allaying fears for patients and specialists. Prog Cardiovasc Dis. 2018;61(3–4):282–6. 30031004 10.1016/j.pcad.2018.07.016

[14] Poamaneagra SC, Plesca DA, Tataranu E, Marginean O, Nemtoi A, Mihai C, et al. A global perspective on transition models for pediatric to adult cystic fibrosis care: What has been made so far? J Clin Med. 2024;13(23):7428. 39685886 10.3390/jcm13237428 PMC11642410

[15] Sechi A, Fabbro E, Langeveld M, Tullio A, Lachmann R, Mochel F, et al. Education and training in adult metabolic medicine: results of an international survey. JIMD Rep. 2019;49(1):63–9. 31497483 10.1002/jmd2.12044 PMC6718119

[16] SIMMESN https://www.simmesn.it/it/.

[17] MetabERN Italia https://metab.ern-net.eu/hcps-and-their-representatives-in-italy/.

[18] REDCap https://www.project-redcap.org/.

[19] Eysenbach G. Improving the quality of Web surveys: the checklist for reporting results of internet E-surveys (CHERRIES). J Med Internet Res. 2004;6(3): e34. 15471760 10.2196/jmir.6.3.e34 PMC1550605

[20] Sirrs S, Hollak C, Merkel M, Sechi A, Glamuzina E, Janssen MC, et al. The frequencies of different inborn errors of metabolism in adult metabolic centres: report from the SSIEM adult metabolic physicians group. JIMD Rep. 2016;27:85–91. 26450566 10.1007/8904_2015_435 PMC5580735

[21] Stepien KM, Kiec-Wilk B, Lampe C, Tangeraas T, Cefalo G, Belmatoug N, et al. Challenges in transition from childhood to adulthood care in rare metabolic diseases: results from the first multi-center European survey. Front Med (Lausanne). 2021;8: 652358. 33738294 10.3389/fmed.2021.652358 PMC7962750

[22] Biasucci G, Brodosi L, Bettocchi I, Noto D, Pochiero F, Urban ML, et al. The management of transitional care of patients affected by phenylketonuria in Italy: Review and expert opinion. Mol Genet Metab. 2022;136(2):94–100. 35589496 10.1016/j.ymgme.2022.04.004

[23] Gold JI, Stepien KM. Healthcare transition in inherited metabolic disorders-is a collaborative approach between US and European centers possible? J Clin Med. 2022;11(19):5805. 36233672 10.3390/jcm11195805 PMC9572070

[24] Sechi A, Urban ML, Murphy E, Pession A, Scarpa M, Group SAMW, et al. Towards needed improvements in inherited metabolic medicine in adulthood: the SIMMESN adult metabolic working group and MetabERN Joint Position Statement. Nutr Metab Cardiovasc Dis. 2024;34(11):2440–5. 39174424 10.1016/j.numecd.2024.07.017

[25] Lampe C, McNelly B, Gevorkian AK, Hendriksz CJ, Lobzhanidze TV, Perez-Lopez J, et al. Transition of patients with mucopolysaccharidosis from paediatric to adult care. Mol Genet Metab Rep. 2019;21: 100508. 31687335 10.1016/j.ymgmr.2019.100508 PMC6819742

