# Supplementary material for: Transition in inherited metabolic diseases: the dietitians, pediatricians and adult physicians’ point of view: the results of an Italian survey
Source: Orphanet J Rare Dis. 2025 May 22;20:241. doi: 10.1186/s13023-025-03755-8 (PMC12100843; doi:10.1186/s13023-025-03755-8)
Supplement: Supplementary file 1 — Additional file 1. [file 13023_2025_3755_MOESM1_ESM.pdf]

Survey MME

Gentile Collega,
di seguito troverà le domande relative alla transizione dei pazienti con MME.
Ogni risposta sarà un importante contributo a questa indagine multicentrica.

Qualora ci fossero dubbi o domande potrà salvare le risposte già confermate (seguendo le apposite indicazioni) e scriverci, per poi riprendere la survey in un secondo momento.

Grazie e buona compilazione!

Affiliazione ______________________

Città ______________________

Regione ______________________

Professione
- ◯ Medico
- ◯ Dietista

Specializzazione
- ◯ Pediatria
- ◯ Neuropsichiatria Infantile
- ◯ Medicina Interna
- ◯ Gastroenterologia
- ◯ Neurologia
- ◯ Psichiatria
- ◯ Endocrinologia
- ◯ Scienza dell'Alimentazione
- ◯ Altro

Specificare ______________________

Qual è la sua area professionale di competenza? (risposta multipla)
- ☐ Nutrizione pediatrica (pediatria generale + MME)
- ☐ Nutrizione adulto (nutrizione adulto generale + MME)
- ☐ Nutrizione Malattie Metaboliche Ereditarie (solo MME pz pediatrici+adulti)
- ☐ Nutrizione Malattie Metaboliche Ereditarie (solo MME pz pediatrici)
- ☐ Nutrizione Malattie Metaboliche Ereditarie (solo MME pz adulti)

Di quale popolazione con MME si occupa? (risposta multipla)
- ☐ Bambini
- ☐ Adulti

Quali delle seguenti malattie metaboliche ereditarie (MME) vengono trattate nel suo Centro? (risposta multipla)	☐ DIFETTI CONGENITI DEL METABOLISMO E DEL TRASPORT DEGLI AMINOACIDI<br>☐ DIFETTI CONGENITI DEL METABOLISMO DEL CICLO DELL'UREA E IPERAMMONIEMIE EREDITARIE<br>☐ DIFETTI CONGENITI DEL METABOLISMO E DEL TRASPORT DEI CARBOIDRATI<br>☐ IPERINSULINISMI CONGENITI<br>☐ DIFETTI CONGENITI DEL METABOLISMO DELLE LIPOPROTEINE<br>☐ DIFETTI CONGENITI DELLA SINTESI DEL COLESTEROLO, DEGLI ACIDI BILIARI e DEI FOSFOLIPIDI E DEI GLICOSFINGOLIPIDI<br>☐ MALATTIE PEROSSISOMIALI<br>☐ DIFETTI CONGENITI DEL METABOLISMO DEI NEUROTRASMETTITORI E DEI PICCOLI PEPTIDI<br>☐ DIFETTI CONGENITI DEL METABOLISMO DELLE PORFIRINI E DELL'EME<br>☐ DIFETTI CONGENITI DEL METABOLISMO DELLE PURINE E DELLE PIRIMIDINE<br>☐ DIFETTI CONGENITI DEL METABOLISMO ENERGETICO MITOCONDRIALE<br>☐ MALATTIE DA ACCUMULO LISOSOMIALE<br>☐ DIFETTI CONGENITI DELL'ASSORBIMENTO E DEL TRASPORTO DI VITAMINE E COFATTORI NON PROTEICI<br>☐ DIFETTI CONGENITI DEL METABOLISMO E DEL TRASPORT DI METALLI<br>☐ DIFETTI CONGENITI DEL METABOLISMO E DEL TRASPORT DELLE PROTEINE
Quanti anni ha il paziente più giovane in follow-up?	________________________________<br>(anni)
Quanti anni ha il paziente più anziano in follow-up?	________________________________<br>(anni)
Nella sua Struttura ospedaliera è in vigore un percorso di transizione mirato alla presa in carico del paziente adulto affetto da MME?	◯ Si, riconosciuto ufficialmente<br>◯ Si ma non riconosciuto ufficialmente<br>◯ No
Da quanti anni è in vigore il percorso di transizione del paziente adulto affetto da MME?	________________________________<br>(anni)
A che età minima è prevista la transizione del paziente con MME, al Servizio dell'adulto?	◯ 16 anni<br>◯ 18 anni<br>◯ Non c'è un'età minima<br>◯ Altro
Specificare	________________________________

A quale Servizio dell'adulto viene fatto transitare il paziente?	◯ ad un'unica struttura/UO dello stesso presidio ospedaliero<br>◯ a più strutture/UO dello stesso presidio ospedaliero<br>◯ ad un'unica struttura/UO di un altro presidio ospedaliero<br>◯ a più strutture/UO di un altro presidio ospedaliero
Specifichi il nome e l'area di specializzazione della/delle struttura/UO:	__________________________________
Da quale Servizio pediatrico viene fatto transitare il paziente?	◯ da un'unica struttura/UO dello stesso presidio ospedaliero<br>◯ da più strutture/UO dello stesso presidio ospedaliero<br>◯ da un'unica struttura/UO di un altro presidio ospedaliero<br>◯ da più strutture/UO di un altro presidio ospedaliero
Specifichi il nome e l'area di specializzazione della/delle struttura/UO:	__________________________________
Da quali figure professionali è composta l'équipe che prende in carico il paziente dopo la transizione? (risposta multipla)	☐ Pediatra<br>☐ Medico/i specialista/i<br>☐ Dietista<br>☐ Infermiera/e<br>☐ Psicologa/o<br>☐ Biologa/o<br>☐ Ostetrica/o<br>☐ Altro
Medico/i specialista/i in:	__________________________________
Specificare:	__________________________________
Quante visite congiunte sono previste prima della totale presa in carico del paziente da parte del Servizio dell'adulto?	__________________________________
Quali figure professionali partecipano alla/e visita/e congiunta/e? (risposta multipla)	☐ Pediatra<br>☐ Medico/i specialista/i<br>☐ Dietista<br>☐ Infermiera/e<br>☐ Psicologa/o<br>☐ Biologa/o<br>☐ Ostetrica/o<br>☐ Altro
Medico/i specialista/i in:	__________________________________
Specificare	__________________________________

Dopo la transizione, all'interno dell'equipe, quale figura professionale segue il paziente adulto con MME dal punto di vista nutrizionale?	◯ Medico (nel Centro non è prevista il/la dietista)<br>◯ Dietista<br>◯ Altro
Specificare	__________________________________
Quale dietista prende in carico i pazienti adulti con MME dopo la transizione?	◯ Stesso/a dietista che segue i pazienti pediatrici con MME (non cambia dopo la transizione)<br>◯ Nuovo/a dietista dedicato ai pazienti adulti con MME<br>◯ Qualsiasi dietista del servizio di nutrizione (es. attivazione consulenza, dietista non dedicato alle MME)<br>◯ Altro
Specificare	__________________________________
E' previsto un passaggio di consegne ?	◯ Si, con incontri programmati<br>◯ Si, solo tramite invio di documentazione da remoto<br>◯ E' il paziente che provvede a portare la propria documentazione al nuovo professionista<br>◯ Non è previsto alcun passaggio di consegne
Quanti pazienti all'anno vengono transitati?	__________________________________
È prevista una valutazione psicologica e sociale sullo stato di preparazione alla transizione?	◯ Yes<br>◯ No
È prevista una valutazione della soddisfazione del paziente/famiglia a qualche mese dall'avvenuta transizione?	◯ Yes<br>◯ No
È prevista una valutazione della QoL del paziente a qualche mese dall'avvenuta transizione?	◯ Yes<br>◯ No

Nella sua esperienza, quali difficoltà o ostacoli vengono riscontrati nel percorso di transizione dal punto di vista logistico-organizzativo? (risposta multipla)	☐ Difficoltà dovute all'invio dei pazienti in un altro presidio ospedaliero (es. cambio di ospedale con diversa dislocazione)<br>☐ Difficoltà nell'accesso ai dati e alla storia clinica dei pazienti<br>☐ Mancanza di uno/una psicologo/a nel Servizio dell'adulto<br>☐ Mancanza di un/una dietista nel Servizio dell'adulto<br>☐ Difficoltà nel passaggio di informazioni tra i team e/o le strutture/UO<br>☐ Il medico pediatra è restio ad affidare il paziente a un altro medico/struttura<br>☐ Mancanza di formazione/esperienza specifica sulle MME del team del Servizio dell'adulto<br>☐ Mancanza di un setting di cura adeguato ai pazienti adulti (es. mancanza di spazi/strumentazioni idonei a pazienti in età adulta)<br>☐ Mancanza di risorse di laboratorio/programmi specifici per il follow-up del paziente (es. mancanza di un laboratorio analisi specializzato, programmi di prenotazione esami..)<br>☐ Altro
Specificare:	__________________________________
Nella sua esperienza, quali difficoltà od ostacoli vengono riscontrati nel percorso di transizione dal punto di vista del paziente e/o dei genitori/caregivers? (risposta multipla)	☐ Il paziente è restio a lasciare la struttura pediatrica e ad accettare un nuovo team<br>☐ I genitori/caregivers sono restii a lasciare la struttura pediatrica e ad accettare un nuovo team<br>☐ Difficoltà a recarsi in un nuovo presidio ospedaliero (es. cambio di ospedale con diversa dislocazione)<br>☐ Il paziente deve responsabilizzarsi nella gestione della propria patologia<br>☐ Il paziente/genitori/caregivers sono restii ad affrontare nuovi aspetti clinici (es. gravidanza..)<br>☐ Mancanza di uno/una psicologo/a nel Servizio dell'adulto<br>☐ Mancanza di un/una dietista nel Servizio dell'adulto<br>☐ Altro
Specificare:	__________________________________
Nella sua esperienza, quali aspetti positivi vengono riscontrati nel percorso di transizione dal punto di vista logistico-organizzativo? (risposta multipla)	☐ Percorso assistenziale facilitato (prenotazione esami, consulenze..)<br>☐ Setting di cura adeguato ai pazienti adulti (es. presenza di spazi/strumentazioni idonei a pazienti in età adulta)<br>☐ Adeguate risorse di laboratorio/programmi specifici per il follow-up del paziente<br>☐ Adeguata gestione delle competenze specialistiche di ciascun professionista (es. il pediatra non si occupa più di pazienti adulti)<br>☐ Adeguato passaggio di informazioni tra i team e/o le strutture/UO e accesso ai dati del paziente<br>☐ Altro

Specificare:	__________________________________
Nella sua esperienza, quali aspetti positivi vengono riscontrati nel percorso di transizione dal punto di vista del paziente e/o dei genitori/caregivers? (risposta multipla)	☐ Setting di cura più adeguato ai pazienti adulti (es. spazi/strumentazioni idonei a pazienti in età adulta)<br>☐ Il paziente/genitori/caregivers possono avere una più specifica presa in carico e affrontare nuovi aspetti clinici (es. gravidanza..)<br>☐ Il paziente può responsabilizzarsi nella gestione della propria patologia<br>☐ Altro
Specificare:	__________________________________
Riuscirebbe a quantificare la percentuale di pazienti divenuti adulti che sospendono il rapporto con la Struttura pediatrica di riferimento?	◯ meno del 10%<br>◯ tra il 10-25%<br>◯ tra il 25-50%<br>◯ oltre il 50%
Quali sono le principali difficoltà nel mantenere in follow-up sia pazienti pediatrici che adulti? (risposta multipla)	☐ Crescente numerosità dei pazienti e del carico di lavoro<br>☐ Minore esperienza sugli aspetti nutrizionali del paziente adulto<br>☐ Mancanza di un medico specialista dell'adulto<br>☐ Organizzazione di percorsi assistenziali specifici per il paziente adulto (terapia enzimatica sostitutiva, gravidanza, ...)<br>☐ Altro
Specificare:	__________________________________
Nella sua esperienza, quali sono i principali aspetti negativi dovuti all'assenza di un percorso di transizione dal punto di vista del paziente e/o dei genitori/caregivers? (risposta multipla)	☐ Setting di cura non adeguato al paziente adulto (es: mancanza di spazi/strumentazioni idonee)<br>☐ Mancanza di una specifica presa in carico per paziente/genitori/caregivers focalizzata su nuovi possibili aspetti clinici (es: gravidanza, insorgenza di nuove patologie, ...)<br>☐ Scarsa/assente responsabilizzazione del paziente nella gestione della propria patologia<br>☐ Altro
Specificare:	__________________________________
Quali pensa siano i principali ostacoli che ad oggi non hanno permesso la realizzazione di un percorso di transizione? (risposta multipla)	☐ Individuazione di una idonea struttura/UO a cui transitare i pazienti (dello stesso o di altro presidio ospedaliero)<br>☐ Mancanza di un team con formazione specialistica sulle MME<br>☐ Mancanza di una o più figure professionali (es. medico/dietista/psicologo/infermieri..)<br>☐ Difficoltà nell'individuare tutte le strumentazioni, spazi, risorse necessarie alla presa in carico<br>☐ Altro

Specificare:

____

È prevista per il futuro l'istituzione di un percorso di transizione ai servizi dell'adulto mirato alla presa in carico del paziente adulto con MME?

- ◯ Si
- ◯ No
- ◯ Attualmente non ne sono a conoscenza

Ritiene che sarebbe necessaria l'istituzione di un percorso di transizione ai Servizi dell'adulto?

- ◯ Si
- ◯ No

Per quali patologie ritiene che sarebbe necessaria l'istituzione di un percorso di transizione?

- ◯ Per tutte le MME
- ◯ Solo per alcune

Quali? (risposta multipla)

- ☐ DIFETTI CONGENITI DEL METABOLISMO E DEL TRASPORT DEGLI AMINOACIDI
- ☐ DIFETTI CONGENITI DEL METABOLISMO DEL CICLO DELL'UREA E IPERAMMONIEMIE EREDITARIE
- ☐ DIFETTI CONGENITI DEL METABOLISMO E DEL TRASPORT DEI CARBOIDRATI
- ☐ IPERINSULINISMI CONGENITI
- ☐ DIFETTI CONGENITI DEL METABOLISMO DELLE LIPOPROTEINE
- ☐ DIFETTI CONGENITI DELLA SINTESI DEL COLESTEROLO, DEGLI ACIDI BILIARI e DEI FOSFOLIPIDI E DEI GLICOSFINGOLIPIDI
- ☐ MALATTIE PEROSSISOMIALI
- ☐ DIFETTI CONGENITI DEL METABOLISMO DEI NEUROTRASMETTITORI E DEI PICCOLI PEPTIDI
- ☐ DIFETTI CONGENITI DEL METABOLISMO DELLE PORFIRINI E DELL'EME
- ☐ DIFETTI CONGENITI DEL METABOLISMO DELLE PURINE E DELLE PIRIMIDINE
- ☐ DIFETTI CONGENITI DEL METABOLISMO ENERGETICO MITOCONDRIALE
- ☐ MALATTIE DA ACCUMULO LISOSOMIALE
- ☐ DIFETTI CONGENITI DELL'ASSORBIMENTO E DEL TRASPORTO DI VITAMINE E COFATTORI NON PROTEICI
- ☐ DIFETTI CONGENITI DEL METABOLISMO E DEL TRASPORT DI METALLI
- ☐ DIFETTI CONGENITI DEL METABOLISMO E DEL TRASPORT DELLE PROTEINE

A quale età minima ritiene che dovrebbe essere fatto transitare il paziente?

- ◯ 16 anni
- ◯ 18 anni
- ◯ Non c'è un'età minima
- ◯ Altro

Specificare:

____

A quale/i struttura/UO pensa potrebbero essere transitati i pazienti adulti con MME?

- ◯ ad un unica struttura/UO dello stesso presidio ospedaliero
- ◯ a più strutture/UO dello stesso presidio ospedaliero
- ◯ ad un unica struttura/UO di un altro presidio ospedaliero
- ◯ a più strutture/UO di un altro presidio ospedaliero

Una volta avvenuta la transizione, chi ritiene che dovrebbe seguire i pazienti adulti dal punto di vista nutrizionale?	◯ Stessa/o dietista che segue i pazienti pediatrici con MME<br>◯ Nuova/o dietista dedicato ai pazienti adulti con MME<br>◯ Qualsiasi dietista del servizio di nutrizione clinica (es: attivazione consulenza, dietista non dedicata/o alle MME)<br>◯ Altro
Specificare:	__________________________________
